# Static Foot Disturbances and the Quality of Life of Older Person with Rheumatoid Arthritis

**DOI:** 10.3390/ijerph19148633

**Published:** 2022-07-15

**Authors:** Katarzyna Kaniewska, Anna Kuryliszyn-Moskal, Anna Hryniewicz, Diana Moskal-Jasińska, Mariusz Wojciuk, Zofia Dzięcioł-Anikiej

**Affiliations:** 1 Department of Rehabilitation, Medical University of Bialystok, 24A M. Skłodowskiej-Curie St., 15-276 Bialystok, Poland; anna.kuryliszyn-moskal@umb.edu.pl (A.K.-M.); anna.hryniewicz@umb.edu.pl (A.H.); mariusz.wojciuk@umb.edu.pl (M.W.); zofia.dzieciol-anikiej@umb.edu.pl (Z.D.-A.); 2 Department of Clinical Phonoaudiology and Speech Therapy, Medical University of Bialystok, 37 Szpitalna St., 15-276 Bialystok, Poland; diana.moskal-jasinska@umb.edu.pl

**Keywords:** rheumatoid arthritis, frailty syndrome, the aging process, pain, foot statics, AIMS-2

## Abstract

Disturbed static foot function is one of the main causes of impaired quality of life, which may be related to the frailty syndrome of older adult patients with Rheumatoid Arthitis (RA). The aim of the study was to evaluate the relationship between parameters of static foot function disturbances and quality of life of older adult patients with RA. The study was performed among 102 patients with RA diagnosed according to the American College of Rheumatology (ACR) and EULAR 2010 criteria. Patients were divided into four subgroups depending on radiological evaluation according to the Steinbrocker classification. Plantoconturography examination was conducted using a podoscope with a 3D scanner and software for computer foot examination CQ ST2K. Quality of life of patients with RA was evaluated using the Arthritis Impact Measurement Scales-2 (AIMS-2). A statistically significant relationship between AIMS-2 and parameters of static foot function disturbances was observed. The study revealed correlations between parameters of disturbed static foot function and RA severity in comparison to disease duration. Our results indicate a relationship between static foot function disturbances and quality of life of patients with RA, not only in the area of physical activity, but also in the social an emotional domain. Study results indicate that plantoconturography and assessment of quality of life using AIMS-2 could be useful as a diagnostic and prognostic tool in RA.

## 1. Introduction

Rheumatoid arthritis (RA) is a chronic inflammatory disease of the joints. RA mainly affects the small joints of the hands and feet. In the foot, it provokes deformities in the forefoot and hindfoot. These pathological changes affect the joints and ligaments, limiting movement in the ankle and the foot. It also produces an unequal distribution of pressure, making it painful to remain in a standing position. Foot and ankle problems are especially common in patients with RA, causing significant disability and limitation in daily activities. Improving quality of life of rheumatoid patients is the prime therapeutic goal for medical doctors and physiotherapists. The development of inflammation and progression of pain influence both the physical and psychological functional status of patients. RA is associated with chronic pain which results in a decreased level of physical activity and frequently, the necessity faced by patients to change their life plans. Disease progression may lead to unemployment, social marginalization, economic dependence and even poverty. Patients with dysfunctions resulting from progressing RA are characterised by a significant degree of disability. Clinical studies reveal far lower quality of life in patients with RA compared to healthy individuals [1,2].

Increasingly often, quality of life reported by patients with RA provides valuable information on the effectiveness of pharmacotherapy, rehabilitation and nursing care. The evaluation of treatment outcomes from the perspective of patient quality of life allows for assessment of the long-term effectiveness of treatment for chronic diseases, including RA.

In recent years, frailty, defined as a “a biologic syndrome of decreased reserve and resistance to stressors, resulting from cumulative declines across multiple physiologic systems, and causing vulnerability to adverse outcomes” has emerged as a significant area of research in rheumatology. Frailty is a geriatric syndrome that results from a multi-system reduction in reserves, deterioration of the ability to adapt to stressful situations, and thus an increased risk of such adverse phenomena as infections, falls, deterioration of cognitive abilities, and dependence on other people or institutions. The presence of frailty increases the risk of a more severe course of certain diseases or predisposes to the development of additional health problems [3]. Frailty is closely linked to musculoskeletal health. The overall prevalence of frailty in Europe is assessed as 7.7% in the population aged above 50 years [4]. Musculoskeletal functioning is a key component on quantification of frailty, at the same time, frailty is associated with the most common age-related disease conditions such as rheumatoid arthritis (RA). Advancing research into the determinants of weakness in RA of the determinants of frailty in RA are necessary because, even as the therapeutic armamentarium for RA continues to grow, individuals with RA continue to commonly experience physical disability and reduced health-related quality of life [5,6,7]. Inflammatory disorders and deformities, characteristic of RA, cause abnormalities in the morphology, anatomy and biokinematics of the foot. Progressing inflammatory changes in the foot are associated with hypertrophy of the synovial membrane, relaxation of ligaments and abnormal muscle function, resulting in deformity. Furthermore, enzymatically induced damage to cartilage, articular tissues and bone structures plays an important role in this process. Studies indicate that foot problems affect 55–90% of individuals with RA [8,9,10,11]. Approximately 20% of patients with early stages of RA report symptoms of impaired functionality of feet and ankle joints [12]. It is also worth noting that in 36% of cases, the first symptoms reported by patients concern feet [13].

Abnormal foot biomechanics in patients with RA leads to deformities. Pathologies affect all anatomical parts of the foot: the forefoot, metatarsus, hindfoot and the ankle joint. According to the available literature, most deformities occur within the forefoot [8,9,10,11]. Foot deformities diagnosed in patients with RA include varus of the first metatarsal, valgus of the fifth metatarsal, subluxation in metatarsophalangeal joints, hammer toes, stiff toes, pes planus, adduction and pronation of the forefoot, hypermobile Chopart joint and hindfoot valgus. These anomalies are accompanied by subcutaneous bursitis.

At present, the degree of deformity is evaluated in clinical studies using a modified electronic version of the podoscopic test. Plantocontourography, which offers a computer-aided analysis of parameters, is characterised by higher reliability and thus repeatability. Other advantages of computer-aided foot examination include assessment accuracy and ability to monitor degenerative changes, which are crucial to the development of an appropriate treatment plan. This examination enables not only foot function assessment, but also detection and graphical documentation of biomechanical anomalies in the foot. Plantocontourography offers an accurate representation of the plantar surface and provides detailed information on the three-dimensional structure of the foot arch [6,10,11].

To the best of our knowledge there is a limited number of studies investigating the impact of foot problems on the quality of life of patients with RA [8,9,10,11,12,13]. It seems interesting to estimate if there is a relationship between parameters of static foot function assessed by plantocontourography and the quality of life of older adult patients with RA?

## 2. Materials and Methods

The aim of the study was to evaluate the relationship between parameters of static foot function assessed by plantocontourography and the quality of life of older adult patients with RA.

The study included 102 patients with RA diagnosed according to the American College of Rheumatology (ACR) and the 2010 European League Against Rheumatism (EULAR) criteria [14]. All patients underwent a medical evaluation, including a physical and clinical examination. Particular attention was paid to gait disorders and problems within foot joints reported by patients. Patients with comorbidities affecting static foot function such as diabetes, discopathy and cardiovascular diseases were excluded from the study. Other exclusion criteria were post-traumatic and postoperative conditions in the lower extremities. Due to the procedure of the computer-aided foot examination, patients with RA who were unable to stand without assistance were also excluded. Treatment included methotrexate (MTX) 15–20 mg once a week and folic acid 15 mg once a week (given on the day after MTX administration). Steroids and immunosuppressive drugs were not used during the two months prior to the study.

Patients were divided into four subgroups depending on the radiological evaluation of RA (the Steinbrocker classification). The Disease Activity Score-28 for Rheumatoid Arthritis (DAS-28) was assessed [15]. 

All patients were informed about the nature of the study and its purpose. Patients gave written informed consent for study participation. The study was conducted in accordance with the protocol approved by the Bioethics Committee of the Medical University in Bialystok (No. 114—09904P). All methods were carried out in accordance with relevant guidelines and regulations. This study was performed under the Strengthening the Reporting of Observational Studies in Epidemiology (STROBE) guidelines (Table S1).

Our study was conducted by one researcher. The computer-based plantocotourographic examinations were performed first, then the patients completed the questionnaire assessing the quality of life. 

**Figure 1:**
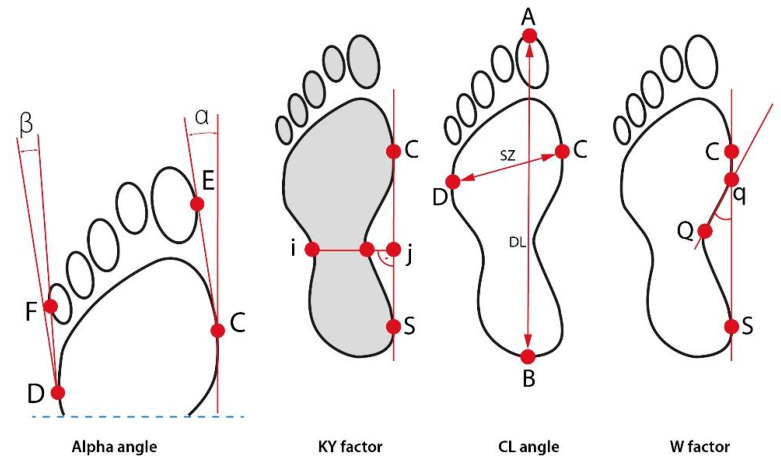
Parameters determining foot deformities. α-hallux valgus angle; β-little toe angle, A-front edge of the foot; B-rear edge of the foot; C-medial edge of the foot; D-lateral edge of the foot; E-outside edge of the hallux; F-outside edge of the little toe, i-lateral edge of plantar contourograph, j-medial edge of plantar contourograph; S- medial edge of the hindfoot; Q, q-internal points of the foot, DL-foot length; SZ-foot width.

The evaluation of static foot function was performed using a podoscope with a 3D scanner and software for computer-aided foot examination CQ ST2K (CQ—Stopy USB v.2011.09 for WinXP/7, Świerc A., CQ Elektronic System, Czernica, Poland). The operation of this device relies on photogrammetry which consists in performing anthropometric calculations based on a photograph of the examined surface. Characteristic points on the feet were established and used for the calculation of parameters such as the hallux valgus alpha angle (α), Clarke angle (CL), Wejsflog index (W) and Sztriter-Godunow index (KY). (Figure 1) The following parameters, indicating foot deformity, were determined: hallux valgus angle (α) (the angle between a tangential line of the medial edge of the foot and a tangential line from the widest part of the forefoot to the outside edge of the hallux). The norm is 0–9°. The Sztriter-Godunow factor (KY) represents the proportion of the length of the segment located in the foot arch centre (blackened area) and the length of the segment marked by the unblackened part of the plantar contourograph, [KY = (W − i)/(j − I)]. The Wejsflog index (W) estimates the proportion between the length and width of the foot. Its normal proportion is 3:1. Clarke’s angle (CL)—measurement of this index involves marking a straight line that joins the most internal points of the forefoot and the rear foot and internal line (Q-q). The interpretation of the value of Clarke’s angle: flat foot ≤30°, foot with a lower arch 31–41°, foot with a normal arch 42–54°, foot with a higher arch ≥55° (Figure 1) [9,10,11,16,17].

The quality of life of patients with RA was assessed using the Arthritis Impact Measurement Scales-2 (AIMS-2), a shorter version of AIMS, developed by Boston University School of Public Health. The AIMS-2 instrument is a 78-item questionnaire. The first 57 items are broken down into 12 scales assessing mobility level, walking and bending, hand and finger function, arm function, self-care, household tasks, social activities, support from family and friends, arthritis pain, work, level of tension and mood [18]. 

### Statistical Analysis

Statistical analysis assessed the normality of distribution by means of the Kolmogorov-Smirnov test with Lilliefors correction and the Shapiro-Wilk test. The analysed quantitative variables did not exhibit normality of distribution. The non-parametric Mann-Whitney U test was applied in two groups in order to compare quantitative variables without normality of distribution. Spearman’s rank correlation coefficient was also established. The non-parametric ANOVA Kruskal-Wallis test was used to analyse differences in results among the groups. Results were considered to be statistically significant for *p* < 0.05. Calculations were performed using the Statistica 10.0 package by StatSoft.

## 3. Results

The study included 102 patients with RA who were in remission (DAS-28 < 2.6). Table 1 shows characteristics of study participants. There were no statistically significant differences between groups.

### 3.1. Inter-Group Differences in Static Foot Function Depending on the Radiological Classification of RA according to Steinbrocker Classification

There were significant inter-group differences in the α angle of the left foot (*p* = 0.04) depending on RA severity. The study revealed a positive correlation between disease severity evaluated according to the Steinbrocker functional classification and the incidence of hallux valgus (α > 9°) (*p* = 0.04). Incidence rates for hallux valgus in both feet in patients with RA Class II and subjects with RA Class III were comparable. In patients with RA Class IV, hallux valgus most commonly affected the right (75%) and left (93%) foot.

The study revealed that impaired static foot function becomes evident in patients with early-stage RA (Class I) and persists, almost unaltered, throughout subsequent stages of the disease.

### 3.2. Inter-Group Differences in the Quality of Life Evaluated with AIMS-2 Depending on the Severity of Radiological Changes (the Steinbrocker Classification) and Duration of RA

#### 3.2.1. Domain: Mobility

**Figure 2:**
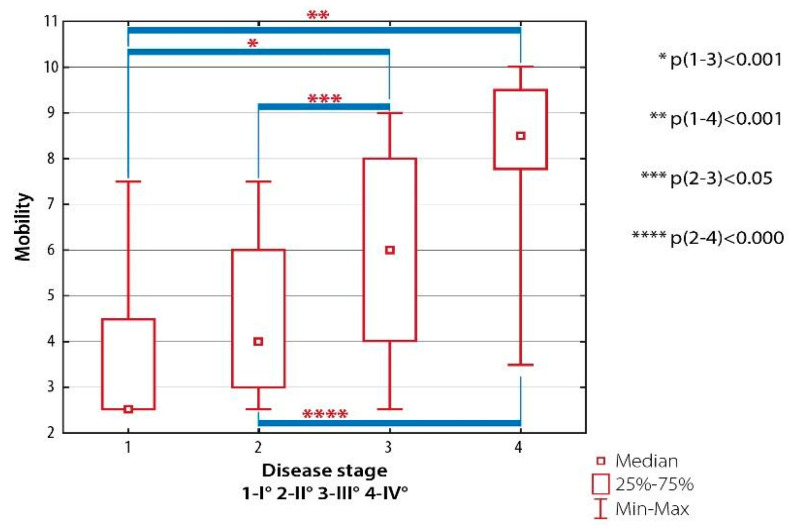
Inter-group differences in the quality of life evaluated with AIMS-2: mobility domain, depending on the severity of radiological changes (the Steinbrocker classification).

There were significant differences in mobility scores between patients with RA Class I and subjects with RA Classes III (*p* < 0.001) and IV (*p* < 0.001), and between patients with RA Class II and subjects with RA Classes III (*p* < 0.005) and IV (*p* < 0.000) (Figure 2).

#### 3.2.2. Domain: Walking and Bending

**Figure 3:**
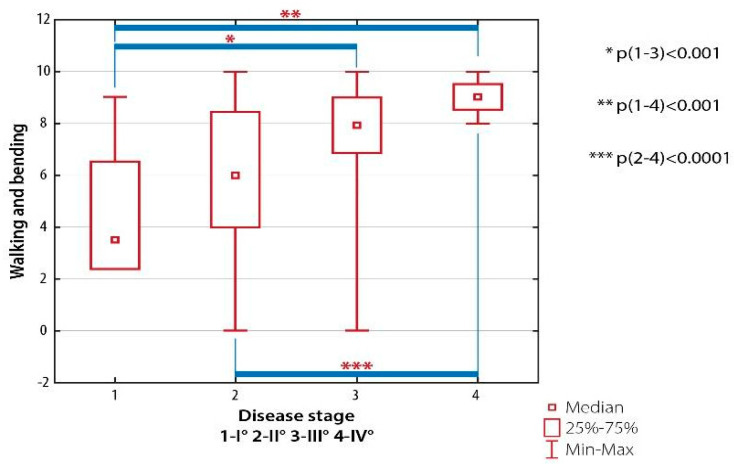
Inter-group differences in the quality of life evaluated with AIMS-2: walking and banding domain, depending on the severity of radiological changes (the Steinbrocker classification).

There were significant differences in walking and bending scores between patients with RA Class I and subjects with RA Class III and IV (*p* < 0.001) as well as between patients with RA Class II and subjects with RA Class IV (*p* < 0.001) (Figure 3).

#### 3.2.3. Domain: Self-Care Tasks

**Figure 4:**
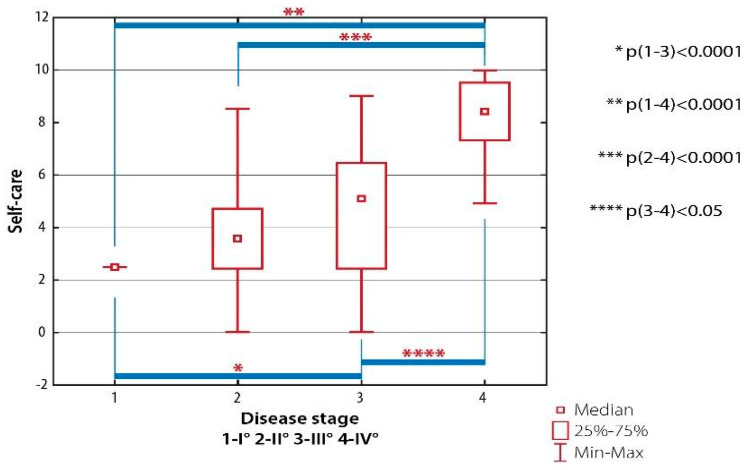
Inter-group differences in the quality of life evaluated with AIMS-2: self-care domain, depending on the severity of radiological changes (the Steinbrocker classification).

There were significant differences in the level of physical activity related to self-care tasks between patients with RA Class I and subjects with RA Classes III (*p* < 0.001) and IV (*p* < 0.001), between patients with RA Class II and individuals with RA Class IV (*p* < 0.001) as well as between patients with RA Class III and subjects with RA Class IV (*p* < 0.005) (Figure 4).

#### 3.2.4. Domain: Household Tasks

**Figure 5:**
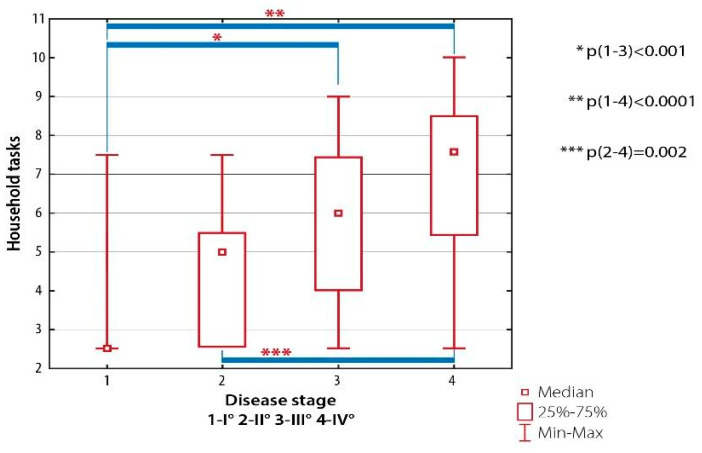
Inter-group differences in the quality of life evaluated with AIMS-2: household tasks, depending on the severity of radiological changes (the Steinbrocker classification).

There were significant differences between patients with RA Class I and those with RA Classes III (*p* < 0.001) and IV (*p* < 0.001) as well as between patients with RA Class II and subjects with RA Class IV (*p* < 0.002) (Figure 5).

#### 3.2.5. Domain: Arthritis Pain

**Figure 6:**
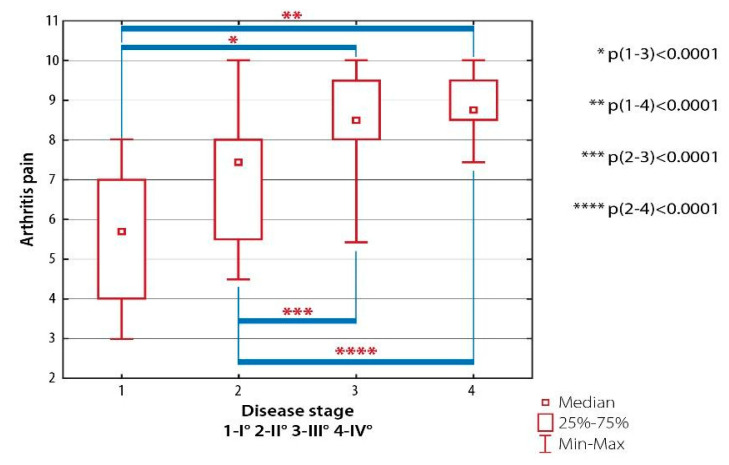
Inter-group differences in the quality of life evaluated with AIMS-2: arthritis pain, depending on the severity of radiological changes (the Steinbrocker classification).

Significant differences were established between patients with RA Class I and those with RA Classes III (*p* < 0.001) and IV (*p* < 0.001) as well as between patients with RA Class II and subjects with RA Classes III (*p* < 0.001) and IV (*p* < 0.001) (Figure 6).

### 3.3. Inter-Group Differences in Emotional Well-Being Scored with AIMS-2 Depending on the Radiological Classification of RA

**Figure 7:**
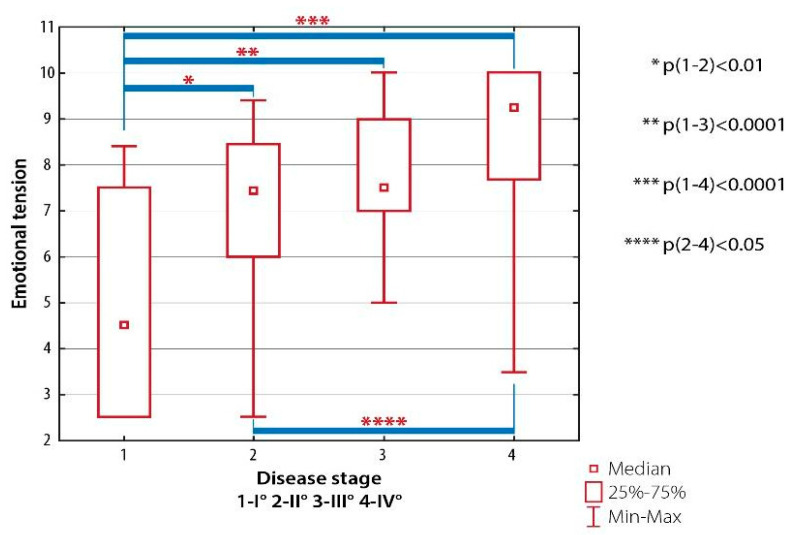
Inter-group differences in emotional well-being scored with AIMS-2: emotional tension, depending on the radiological classification of RA (the Steinbrocker classification).

There were significant differences in the level of emotional tension between patients with RA Class I and those with RA Classes II (*p* < 0.01), III (*p* < 0.001) and IV (*p* < 0.001) as well as between patients with RA Class II and subjects with RA Class IV (*p* < 0.05) (Figure 7). 

**Figure 8:**
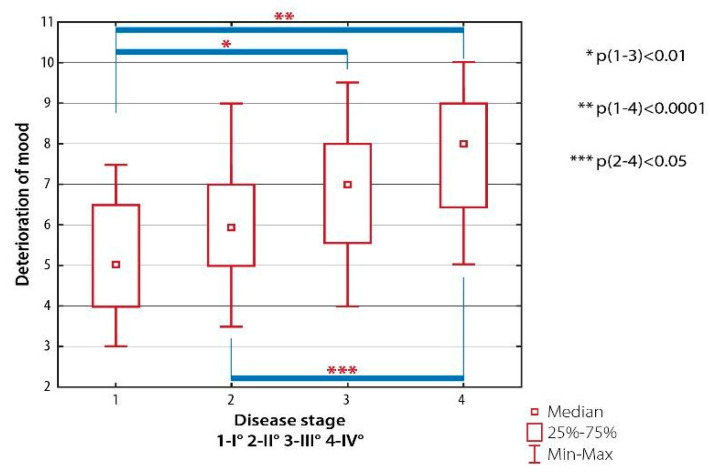
Inter-group differences in emotional well-being scored with AIMS-2: deterioration of mood, depending on the radiological classification of RA (the Steinbrocker classification).

Deterioration of mood correlated with duration of RA (Figure 8).

## 4. Discussion

Rheumatoid arthritis is one of the most common systemic connective tissue disorders. In Poland RA affects approximately 1% of the adult population, which is around 400,000 individuals [19,20]. Recent medical studies reflect an increased interest in health-related quality of life of patients, particularly those with chronic diseases [21,22,23].

Measures designed to address problems limiting patient quality of life, including conditions referred to as the rheumatoid foot have been examined in many studies [9,10,11]. 

The relationship between frailty and RA is not yet fully characterised but these conditions share many of the same clinical outcomes, associations and suggested pathophysiology. It may be seen that the RA and frailty, are closely related, both are multidimensional concept characterised by deficits in multiple organ systems, i.e., psychological, cognitive, and/or social support and other environmental factors, as well as physical limitations. 

Bak et al. in their study concluded that frailty syndrome has no significant impact on the quality of life of patients with diagnosed RA [24].

However, there are no studies on the impact of disturbances in foot morphology on the occurrence of the frailty syndrome

Studies conducted among patients with RA indicate an insufficient level of knowledge regarding foot care and prevention of pathological changes in the foot. Implementation of educational programmes on healthy behaviours, including learning correct movement patterns, improving accessibility in buildings, and use of mobility aids and equipment determine increased treatment effectiveness [25,26,27]. 

Functional foot problems affect approximately 10–24% of the population and are more common in geriatric patients and those suffering from RA [10,11,28,29]. 

Interestingly, Williams et al. reported that the majority of patients with RA complain of foot problems even before the diagnosis of RA is established. Foot deformity leads to a partial or, sometimes, total loss of mobility in foot joints and pain, commonly located in the forefoot. Unfortunately, studies have revealed that in many cases this problem is underestimated by family doctors [30,31].

Foot deformities associated with RA reflect disorders of static foot function. Pathological changes in the rheumatoid foot are considered the most common causes of disability [32,33].

Foot deformities are not just an aesthetic problem, but a cause of persistent pain which reduces patient quality of life. Within the first four years of diagnosis, as many as 75% of patients present with nagging pain which prevents them from being active in many areas of life [34]. Our study revealed differences in AIMS-2 scores on the “walking and bending” scale depending on severity of RA. Our results demonstrate a gradual deterioration in mobility associated with the progression of RA. Other authors have reported a correlation between gait disorders and changes in the radiographic image of the metatarsophalangeal joints. Moreover, the authors emphasised that, most frequently, gait disorders progress rapidly in the early stages of RA and stabilize a year after disease onset [35].

In the present study, evaluation of abnormal static foot function in patients with RA was based on the hallux valgus alpha angle (α), the Clarke angle (CL) and the Wejsflog index (W).

In our investigation, statistical analysis of individual parameters of computer-aided plantoconturography examination revealed similar correlations. We established correlations between parameters of static foot function and quality of life depending on the Steinbrocker classification of RA. The study demonstrated that static foot disorders had a significant impact on all areas of life evaluated with AIMS-2 in patients with RA Class I and RA Class IV. In RA Class II, a correlation was established between the value of the α angle and the AIMS-2 score for household tasks. In patients with RA Class III, the only correlation that was observed regarded the value of the CL angle and the AIMS-2 score for self-care tasks. 

Apart from the biological aspects of health and well-being, clinical studies focus on patients’ emotional state and their ability to lead a normal life. Patient self-assessment reports provide information on limitations encountered in specific areas of life and the possibility of learning new behaviours in various situations related to the progression of RA [36]. When the diagnosis of RA is established, patients are informed that a complete recovery is not possible and therapeutic success consists in prolongation of life and improvement in the patient’s functional status and quality of life.

Recent studies evaluating the quality of life of rheumatoid patients rely on specific measurement tools. Combination, disease-specific questionnaires which can be customised to a given study are used [37]. The suitability of combination questionnaires is emphasised by a number of authors [38,39,40]. A meta-analysis by Matcham et al. revealed that RA has a significant impact on the assessment of health-related quality of life. The authors highlighted the fact that the majority of studies demonstrate a more destructive impact of progressing RA on the mobility of patients than on their emotional state [38]. 

The quality of life of rheumatoid patients is also determined by their age. El-Labban et al. reported a higher degree of physical disability in patients younger than 60 years compared to those older than 60. Moreover, the authors observed greater prevalence of small joint arthritis of the hand and foot in younger people, which affects their ability to perform self-care tasks [41]. The present study did not demonstrate any significant age-related differences between individual subgroups depending on RA severity (the Steinbrocker classification). 

Squire used an open interview to analyse the impact of RA on the professional life of patients. The author revealed that patients need to adapt to perform motor activities depending on their functional status and disease duration. Due to disease progression, patients frequently face the necessity of changing their workplace and type of occupation. Nevertheless, the study demonstrated that being able to pursue a professional career has a positive effect on self-esteem and the emotional status of patients [42].

Other studies indicate numerous difficulties resulting from the absence of standardised methods of documenting and reporting foot health assessment data, monitoring the joint degeneration process and planning treatment [43,44].

Williams et al., who are members of the North West Clinical Effectiveness Group for the Foot in Rheumatic Disease (NWCEG), point to the need to establish gold standards for management of patients with foot deformities and emphasise the role of a multidisciplinary team that should be involved in the treatment process. The authors draw particular attention to the prevention of foot deformities at an early stage of RA development. Furthermore, they underline the importance of special measurement tools such as the foot function index (FFI) [45]. The incorporation of questionnaires assessing quality of life in the treatment of patients with RA allows for direct involvement of the patient in the decision-making process regarding therapy, which can improve its outcomes [46,47].

A number of studies have evaluated the effectiveness of pharmacological, surgical and physiotherapy treatment based on the assessment of quality of life of rheumatoid patients [27]. Quality of life questionnaires have also been used to assess the effectiveness of orthotic treatment in patients with RA. Studies demonstrate an improvement in foot function in patients who use orthopaedic appliances for the forefoot [8,47]. There are no detailed reports in the available literature on the correlation between parameters of static foot function and the quality of life of patients with RA. A limited number of studies have evaluated the relationship between parameters of plantocontourography and the quality of life of patients with RA. The present study demonstrated correlations between parameters of static foot function and the domains of physical activity, social interactions and emotional well-being, assessed with AIMS-2 in groups of patients with different severity of RA. 

One of the few studies investigating the correlation between the clinical status of feet in patients with RA and quality of life was conducted by Otter et al. The authors of the study designed their own questionnaire to assess the impact of foot problems on the quality of life of 390 patients with RA. Almost all surveyed patients reported a negative effect of foot dysfunction on self-perceived quality of life. Impaired mobility caused by foot deformities is a key factor that is directly correlated with limitations in the social domain. Study participants reported greatest problems with mobility and choice of footwear. A correlation was found between quality of life and clinical symptoms such as pain, swelling, stiffness and numbness. Moreover, foot dysfunctions caused a lowering of mood in patients due to significantly limited involvement in social activities. Patients also emphasised the relationship between foot deformities and sleep quality [47].

Unfortunately, our study has some limitations such as: too low number of patients with RA and absence of control group.

## 5. Conclusions

Identifying correlations between the physical, emotional and social status of patients with RA and static foot disorders as well as functional limitations highlights the fundamental role of the assessment of patient quality of life.

Prevalence and knowledge of factors that influence frailty in rheumatic diseases, that is connected with in foot deformities need to be develop.

Furthermore, findings from the above-mentioned studies indicate the need for a plantocontourography examination and the use of tools for assessing patient quality of life (e.g., AIMS-2), and their incorporation in the diagnostic process of RA. These procedures appear to be helpful in establishing therapeutic standards that include both medical and social support.

Plantoconturography is a perfect complement to the standard diagnostics in RA. The results of the conducted research suggest that the plantoconturography test could be useful in diagnosis, prognosis of the course of the disease, and start at the right moment with physiotherapeutic intervention.

## Figures and Tables

**Table 1:** Age and anthropometric measurements of patients with RA depending on disease severity according to the Steinbrocker classification.

	Class I RA n = 18	Class II RA n = 37	Class III RA n = 31	Class IV RA n = 16
**Disease duration (years)**	6.33 ± 5.83	9.59 ± 6.92	17.32 ± 7.28	22.68 ± 8.84
**Age (years)**	65.16 ± 5.85	67.00 ± 4.82	70.06 ± 2.35	69.93 ± 4.19
**Weight (kg)**	65.83 ± 11.64	72.67 ± 14.09	69.54 ± 12.25	71.5 ± 12.63
**Height (m)**	1.63 ± 0.07	1.63 ± 0.07	1.63 ± 0.07	1.63 ± 0.07
**BMI (kg/m^2^)**	24.80 ± 4.6	27.13 ± 4.34	26.08 ± 3.97	26.81 ± 4.1

n—number of patients.

## Data Availability

Datasets used and/or analysed during the study are available from the corresponding author on reasonable request.

## Supplementary Materials

The following supporting information can be downloaded at: https://www.mdpi.com/article/10.3390/ijerph19148633/s1, Table S1: STROBE Statement—checklist of items that should be included in reports of observational studies.
